# Enhancing sweet potato (*Ipomoea batatas*) resilience grown in cadmium-contaminated saline soil: a synergistic approach using *Moringa* leaf extract and effective microorganisms application

**DOI:** 10.1007/s11356-024-33295-w

**Published:** 2024-04-23

**Authors:** Abdelsattar Abdelkhalik, Nasr M. Abdou, Mohammad A. H. Gyushi, Ahmed Shaaban, Shimaa A. Abd El-Mageed, Khaulood A. Hemida, Taia A. Abd El-Mageed

**Affiliations:** 1 Horticulture Department, Faculty of Agriculture, Fayoum University, Fayoum, Egypt; 2 Soil and Water Department, Faculty of Agriculture, Fayoum University, Fayoum, Egypt; 3 Agronomy Department, Faculty of Agriculture, Fayoum University, Fayoum, Egypt; 4 Botany Department, Faculty of Science, Fayoum University, Fayoum, Egypt

**Keywords:** Biostimulants, Salinity, Growth and productivity, Osmoprotectants, Heavy metals, Phytotoxicity

## Abstract

Raising soil contamination with cadmium (Cd^2+^) and salinization necessitates the development of green approaches using bio-elicitors to ensure sustainable crop production and mitigate the detrimental health impacts. Two field trials were carried out to study the individual and combined effects of foliage spraying of *Moringa* leaf extract (MLE) and soil application of effective microorganisms (EMs) on the physio-biochemical, osmolytes, antioxidants, and performance of sweet potato grown in Cd^2+^-contaminated salty soil (Cd^2+^  = 17.42 mg kg^−1^ soil and soil salinity ECe = 7.42 dS m^−1^). Application of MLE, EMs, or MLE plus EMs significantly reduced the accumulation of Cd^2+^ in roots by 55.6%, 50.0%, or 68.1% and in leaves by 31.4%, 27.6%, or 38.0%, respectively, compared to the control. Co-application of MLE and EMs reduced Na^+^ concentration while substantially raising N, P, K^+^, and Ca^2+^ acquisition in the leaves. MLE and EMs-treated plants exhibited higher concentrations of total soluble sugar by 69.6%, free proline by 47.7%, total free amino acids by 29.0%, and protein by 125.7% compared to the control. The enzymatic (SOD, APX, GR, and CAT) and non-enzymatic (phenolic acids, GSH, and AsA) antioxidants increased in plants treated with MLE and/or EMs application. Applying MLE and/or EMs increased the leaf photosynthetic pigment contents, membrane stability, relative water content, water productivity, growth traits, and tuber yield of Cd^2+^ and salt-stressed sweet potato. Consequently, the integrative application of MLE and EMs achieved the best results exceeding the single treatments recommended in future application to sweet potato in saline soil contaminated with Cd^2+^.

**Supplementary Information:**

The online version contains supplementary material available at 10.1007/s11356-024-33295-w.

## Introduction

Salinity and heavy metal-induced soil contamination pose substantial challenges, leading to a major decline in soil quality and agricultural productivity (dos Santos et al. 2022). In addition, salinity stress has an inhibitory effect on plant growth (Abou-Sreea et al. 2022; Zaki and Radwan 2022). This plant growth inhibition in response to salinity may refer to (i) reduced water availability to plants due to increasing osmotic pressure in the soil solution, inducing physiological drought (Semida et al. 2021b), (ii) increased concentration of sodium and chloride ions in saline soils which has a toxic effect on plant growth (Abou-Sreea et al. 2022). Therefore, salinity induces the generation of reactive oxygen species (ROS), which cause oxidative damage, including disruption of protein synthesis, impairment to plasma membranes and cell organelles, suppression of the photosynthetic process, reduction in respiration and gas exchange (Chourasia et al. 2021; Abd El-Mageed et al. 2023). The acquisition of macro- and micro-nutrients by plants is closely linked with the accumulation of Na^+^ ions surrounding plant roots, which significantly alters all ionic homeostasis, causing nutritional deficiency (Abd El-Mageed et al. 2022a, b; Abd El-Mageed et al. 2022a, b).

Heavy metal-contaminated soils have seriously increased due to the rapid development of industrialization, long-term waste recycling, excessive use of chemical products, and irrigation with sewage (Li et al. 2019; Abd El-Mageed et al. 2020). Cadmium (Cd^2+^) is a toxic heavy metal not necessary for plants. Cadmium is characterized by high water solubility and fluidity that accelerates its absorption by the roots of plants, resulting in the suppression of cell division and root elongation (Souid et al. 2020). High Cd^2+^ levels in the soil evoke osmotic stress, restricting water uptake, disturbing ion homeostasis, and inducing nutrient deficiency (Rucińska-Sobkowiak 2016). Moreover, excessive Cd^2+^ concentration causes physiological disorders, including reduced respiration, photoinhibition, decreased water fluidity and gas exchange, the production of chlorophyll, and impairment of the plant’s antioxidant defense mechanism via motivating ROS production (Abd El-Mageed et al. 2019; Genchi et al. 2020; Rasafi et al. 2022).

Under harsh conditions like saline and/or Cd^2+^ contamination, grown plants enhance their machinery tolerance to combat adverse stresses by accumulating osmoregulatory active solutes, including soluble sugars and proline, and elevating their antioxidative capacity (Abd El-Mageed et al. 2020). However, in the majority of cases, the endogenous plant defense mechanisms are insufficient to sustain normal development under stress conditions, necessitating the application of exogenous support, such as the use of biostimulants, to raise their stress tolerance (Ramzan and Younis 2022). Due to increasing soil salinization and contamination with Cd^2+^, developing green strategies using bio-elicitors to reduce the harmful consequences caused by salinity and/or Cd^2+^ stress has generated massive interest worldwide (Batool et al. 2020; Safwat and Matta 2021). Therefore, the current experiment proposed effective microorganisms (EMs) and *Moringa* leaf extract (MLE) as natural biostimulants to mitigate the detrimental impacts on sweet potato plants grown in Cd^2+^-contaminated saline soils.

Effective microorganisms (EMs) are a group of collection cultures comprising favorable micro-organisms, including lactobacilli, photosynthetic bacteria, yeasts, and actinomycetes (Iriti et al. 2019; Khademian et al. 2019). EMs favor soil health by improving soil structure, decomposition of organic matter, accumulation of humus substances, and enhancing the uptake of nutrients, which in turn upgrades soil profitability and sustainability for plant production (Sagar et al. 2021; Abd El-Mageed et al. 2022a, b). The EMs also effectively contribute to improving plant growth by boosting the accessibility and uptake of nutrients, synthesizing metabolites, and producing bioactive molecules, including phytohormones, amino acids, enzymes, nucleic acids, and sugars (Talaat 2019). Therefore, due to its vital effect on plant physio-biochemical responses, using EMs significantly promotes cell division that ensures better germination, development, product quality, and yield of crops in circumstances of stress (Abu-Qaoud et al. 2021). Besides its stimulating effect on plant growth, EMs may also improve the soil’s biological and physicochemical characteristics and have a suppressive effect on pests and soil-borne diseases (De Corato 2021).

A member of the Moringaceae family, *Moringa oleifera* is considered one of the most widespread trees in tropical areas (Gopalakrishnan et al. 2016). The roots and leaves of these trees are regarded as sources of current plant biostimulants (Abd El-Mageed et al. 2017). The *Moringa* leaf extract (MLE) is a naturally rich resource of essential amino acids, carotenoids, flavonoids, proteins, vitamins (A, B_2_, C, and E), ascorbates, phenolics, and numerous nutrients, making it a superior plant growth bio-regulator (Abalaka et al. 2022; Irshad et al. 2022). Further, MLE was shown to be a good source of numerous antioxidants and phytohormones, such as cytokinins (zeatin), auxins, and gibberellins (Abd El-Mageed et al. 2017). The exogenous application of MLE improved plant growth, plant water relations, photosynthetic pigments, photochemical activity, and plant defenses to overcome oxidative stress by ROS scavenging via increasing the production of osmoprotectants and antioxidant enzymes (Khan et al. 2022). Foliar application of MLE on Sudan grass (Desoky et al. 2019a), quinoa (Rashid et al. 2018), wheat (Ahmed et al. 2021), bean (Howladar 2014), and potato (El-Mohamed et al. 2016) resulted in a significant yield increase under different abiotic stress.

Sweet potato (*Ipomoea batatas* L.) is among the Convolvulaceae family cultivated for their valuable tuberous root (Byju and George 2005). Tubers are rich in carbohydrates, especially the orange-fleshed roots, which are an excellent provider of the vitamin A precursor and *β*-carotene (Meng et al. 2020). Worldwide, among food crops, sweet potato occupies the seventh rank. Several reports have shown that Cd^2+^ and salinity stress negatively affected the productivity of sweet potato plants (Meng et al. 2020; Abd El-Mageed et al. 2022a, b).

As plant biostimulants, MLE and EMs are widely used due to their valuable applications and roles in alleviating the detrimental effects of several stresses; however, the information about their coupling application for mitigating the negative effects caused by salinity and Cd^2+^ toxicity on growth and physiological and biochemical responses of sweet potato plants are very limited. Thus, the current research contributes to understanding the role of applied MLE and EMs, alone or in combination, on some physiological and biochemical systems of sweet potato plants against the toxicity of Cd^2+^ and salts. Our research hypothesis was that treating sweet potato plants grown in Cd^2+^-contaminated saline soil with foliar spraying of EMs and soil application with MLE might, singly or collaboratively, alleviate the Cd^2+^ toxic effects through decreasing Cd^2+^ ion uptake and boosting morpho-physio-biochemical aspects and productivity.

## Materials and methods

### Location and agronomic details

Two field experiments were conducted from May to October in the summers of 2020 and 2021 in the Fayoum Governorate, Egypt (29°30′00.6″ N 30°52′32.3″ E). The methodology described by Klute and Dirksen (1986) and Page et al. (1982) was used to determine the main physicochemical properties of the tested soil (Table 1).

**Table 1:** Physicochemical properties of the tested soil

Soil layer (cm)	Particle size distribution					
	Clay (%)		Silt (%)	Sand (%)		Texture
	12.50		13.80	74.70		LS
0–30	BD (g cm^−3^)	Ksat (cm h^−1^)	FC (%)	WP (%)		AW (%)
	1.53.0	1.92	23.20	10.34		10.86
pH	7.80		N (mg kg^−1^ soil)		44.8	
ECe (dS m^−1^)	7.42		P (mg kg^−1^ soil)		4.98	
CEC (cmol kg^−1^)	11.20		K (mg kg^−1^ soil)		35.20	
CaCO3 (%)	4.26		Fe (mg kg^−1^ soil)		2.42	
OM (%)	0.95		Mn (mg kg^−1^ soil)		1.42	
ESP	11.20		Zn (mg kg^−1^ soil)		1.24	
Cd2 + (mg kg^−1^ soil)	17.42		Cu (mg kg^−1^ soil)		0.77	

Abbreviations: *BD* bulk density, *Ksat* hydraulic conductivity, *FC* field capacity, *WP* wilting point, *AW* available water, *OM* organic matter, *ESP*, exchangeable Na + percentage; values are means (*n* = 3)

The soil texture analysis was 72.50% sand, 13.39% silt, and 14.11% clay, and it is considered sandy loam soil. The soil ECe (electrical conductivity) was 7.42 dS m^−1^, showing saline soil. The soil is slightly alkaline in pH (7.80), 17.42 mg kg^−1^ for Cd^2+^, 0.98% for organic matter content, 4.26% for CaCO_3_%, 0.04% for N, available K and P was 56.4, and 5.42 meq 100^–1^ g soil, respectively (Table 1).

Sweet potato (cv. Beauregard) vines of 30 days were relocated on May 2, 2020, and May 10, 2021, in rows of 15 m length and 0.7 m width, on which the vines were transplanted at 0.25 m apart. A drip irrigation system of a single line per row and drippers spaced 0.25 m apart with a 4 L h^−1^ discharge rate were used. The test plot comprises 3 planting rows and covers an area of 31.5 m^2^ (15 m × 2.1 m). The other recommended sweet potato farming techniques were followed exactly as advised.

### Experimental design and treatments

The experimental treatments were laid out in a completely randomized block design and performed in triplicates. The four treatments were control, MLE, EMs, and MLE + EMs. After transplanting, the MLE was sprayed on the leaves thrice at 15, 30, and 45 days. To enable the best possible absorption into the sweet potato plant’s leaves, Tween-20 of 0.1% (v/v) was added to the MLE solution, and the control plants were sprayed with only water. The EMs were applied to sweet potato plants as a soil application three times (10, 20, and 30 days after transplanting).

### Preparation and analysis of *Moringa oleifera* leaf extract (MLE)

When the *Moringa* trees were fully mature, they were harvested, and their fresh leaves were collected. Collected leaves were first frozen for an entire night, then crushed, and filtered twice via filter paper (Whatman No. 1). The resulting solution was refined and then centrifuged for 15 min at 8000 × *g* to separate the supernatant, which was then diluted to create an extraction at a 3% concentration for spraying plant foliage (Abd El-Mageed et al. 2017). The nutritional profile and chemical analysis of MLE are presented in Table S1. Tween 20 (0.1%, v/v) was used as a surfactant to enable the best possible absorption into the sweet potato plant leaves.

### Application of effective microorganisms (EMs)

Five different types of beneficial microorganisms, including fermenting fungi, photosynthetic bacteria, lactic acid bacteria, actinomycetes, and yeast were present in EMs. The mixture was prepared by Egypt’s Ministry of Agriculture and Land Reclamation in Giza, as detailed by Abdelkhalik et al. (2023).

## Measurements

### Osmolyte accumulation assay

Sweet potato dry leaves were used for the extraction and measurement of osmolytes, i.e., total soluble sugars (TSS), total free amino acids (TFA), free proline, and soluble proteins in mg g^−1^ dry weight (DW). The techniques of Bates et al. (1973) were used to measure the free proline content. According to Irigoyen et al. (1992), the soluble sugar content in the leaves was determined using a Bausch & Lomb-2000 Spectrophotometer after extraction with 96% (v/v) ethanol. Using the approach of Wu et al. (2004), the amount of TFA in dried leaves was measured. Bradford (1976) recommended methods were used to quantify soluble proteins.

### Assessment of enzymatic and non-enzymatic antioxidants

Bradford’s (1976) method was used to extract plant tissues as a crude enzyme extract to assess enzymatic and non-enzymatic antioxidants. For enzymatic antioxidants in U mg^‒1^ protein, the superoxide dismutase (SOD) enzyme activity was assessed using the approach of Giannopolitis and Ries (1977). Aebi’s (1984) approach was applied for catalase (CAT) activity quantification. According to Rao et al.’s (1996) method, the ascorbate peroxidase (APX) activity was assessed. After monitoring the GSH-dependent oxidation, the cellular glutathione reductase (GR) was measured per Rao et al.’s (1996) method. For non-enzymatic antioxidants, the phenolic acids (mg g^−1^ DW), reduced glutathione (GSH; µmol g^−1^ FW), and ascorbic acid (AsA; µmol g^−1^ FW) contents in fresh sweet potato leaves were quantified as outlined by Bradford (1976), Griffith (1980), and Mukherjee and Choudhuri (1983), respectively.

### Photosynthetic pigments, plant water status, and membrane integrity

Following the instructions provided by Arnon (1949), chlorophyll “a” (chl *a*), chlorophyll “b” (chl *b*), total chlorophyll, and carotenoids were quantified in mg g^−1^ FW. Fresh leaf samples were homogenized in 50 ml of 80% (v/v) acetone before being centrifuged at 10,000 × g for 10 min. The absorbance of the extract was measured using a UV-160 A UV–vis recording spectrometer (Shimadzu, Kyoto, Japan) at 663, 645, and 470 nm for chlorophyll a, chlorophyll b, and carotenoids, respectively. According to Hayat et al. (2007), relative water content (RWC%) was assessed using the following formula:

$$\mathrm{RWC }(\mathrm{\%})=[(\mathrm{fresh mass}-\mathrm{dry mass})/(\mathrm{turgid mass}-\mathrm{dry mass})]\times 100$$

The membrane stability index (MSI%) was estimated using the method, and the next equation was used for calculation. The following equation was used to calculate the membrane stability index (MSI%), which was determined using the Premachandra et al. (1990) approach.

$${\text{MSI}}(\mathrm{\%})=[1-({\text{C}}1/{\text{C}}2)]\times 100$$

where *C*_1_ is the EC of the solution after heating at 40 °C for 30 min, and *C*_2_ is the EC of the solution after boiling at 100 °C for 10 min.

### Leaf mineral contents

The leaves of sweet potato (*n* = 10) were dried, ground into powder, and then chemically analyzed to assess the amount of N, P, K^+^, Ca^2+^, Na^+^, and Cd^2+^ in g kg^−1^ DW. The N content was quantified utilizing a micro-Kjeldahl equipment (Ningbo Medical Instruments Co., Ningbo, China) using the procedures of AOAC (2000). Molybdenum blue was diluted by 8% (w/v) NaHSO_3_-H_2_SO_4_ and H_2_MoO_7_S and used as standard reagents for the quantification of P, according to Jackson (1973). Also, a PerkinElmer Model 3300 atomic absorption spectrophotometer was used to quantify the Ca^2+^ level. In freeze-dried leaf powder suspension (50 mg), K^+^ and Na^+^ concentrations were measured (Lachica et al. 1973).

### Roots and leaves cadmium (Cd^2+^) content

The Cd^2+^ content in sweet potato leaves and roots (tubers) was determined by spectrophotometry (PerkinElmer Model 3300) according to the Chapman and Pratt (1961) approach.

### Agronomic traits

In both seasons, ten individual plants from each experimental unit were randomly collected at harvest (180 days after planting). Firstly, the leaves number of each plant was counted. A digital planimeter (Planix 7) was used to measure the leaf area (dm^2^ plant^−1^), and the leaf area index was computed by dividing the leaf area per plant by the area those plants occupied. The sweet potato shoot was oven-dried at 70 °C until it reached a constant weight, then recording the dry weight. Fifteen plants from each experimental unit were taken to determine the tuber number plant^−1^, weight for each tuber expressed as tuber weight (g), and tuber weight plant^−1^ (g). Moreover, all sweet potato plants in every experimental plot and the fifteen previously sampled were collected, cleaned, and weighed for estimated tuber yield.

### Statistical analysis

For the two seasons based on RCBD, statistical analysis was performed using the GenStat tool (VSN International Ltd, Oxford, UK). The Fisher least-significant difference test was used to separate the means for each treatment at a *p*-value of ≤ 0.05.

## Results

### Growth traits

Saline soil contaminated with Cd^2+^ significantly diminished the growth characteristics of sweet potato (Table 2). The lowest values of leaves number plant^−1^ (222.1), leaf area plant^−1^ (119.9 dm^2^), leaf area index (2.99), and shoot dry weight (131.3 g) were recorded for control plots as averages in both seasons. However, the applied biostimulants, MLE, EMs, and their mixture (MLE + EMs) showed a beneficial effect on the growth traits of sweet potato (Table 2). Foliarly applied MLE or soil-treated EMs increased the leaves number plant^−1^ (by 88.7 or 72.6%), leaf area (by 98.2 or 46.5%), leaf area index (by 98.2 or 46.5%), and shoot dry weight (by 120.0 or 72.4%) when compared to untreated plants (Table 2). Co-application of MLE and EMs showed significant efficacy on leaf number and shoot dry plant^−1^, exceeding the effects of their individual application; meanwhile, no appreciable differences were found with MLE for leaf area and leaf area parameters in ameliorating stress-induced damages to sweet potato growth. Contextually, applying MLE + EMs significantly elevated the abovementioned growth traits by 98.8, 108.7, 109.0, and 184.8%, respectively, compared to the control (Table 2).

**Table 2:** Influence of *Moringa* leaf extract (MLE) and effective microorganisms (EMs) on the growth attributes of sweet potato grown in Cd2 + -contaminated saline soil in the 2020 and 2021 seasons (as average)

Factors	Leaves no	Leaf area plant^−1^ (dm^2^)	Leaves area index	Shoot DW plant^−1^ (g)
Growing season (GS)				
GSI	344.40 ± 6.4a	187.9 ± 4.32a	4.7 ± 0.42a	243.7 ± 3.4b
GSII	350.00 ± 7.6a	183.83 ± 5.3a	4.5 ± 0.36a	265.6 ± 6.4a
Treatment				
Control	222.1 ± 5.4 d	119.9 ± 3.2 c	2.99 ± 0.35c	131.3 ± 2.2d
MLE	419.1 ± 7.8b	237.6 ± 3.1a	5.93 ± 0.68a	288.9 ± 3.4b
EMs	383.4 ± 6.7c	175.3 ± 3.5b	4.38 ± 0.69b	226.3 ± 3.6c
MLE + EMs	441.5 ± 6.7a	250.2 ± 4.8a	6.25 ± 0.75a	373.9 ± 4.5a
*p*-value				
GS	0.154	0.142	0.113	0.023
Treatment	< 0.001	< 0.001	< .001	< 0.001
CV%	2.20	3.40	3.40	1.80

The various lower-case letters on the bar distinguished the treatments, corresponding to Fisher’s test at *p* ≤ 0.05

### Leaves photosynthetic pigments, tissue water content, and membrane stability

The current investigation showed a noticeable deterioration in plant physiological reactions to adverse conditions created by soil salinity and contamination with Cd^2+^ (Table 3). In this regard, the minimum measured values (as the average of the two seasons) of relative water content (70.48%) and membrane stability index (62.81%) were given for non-ameliorated sweet potato plants (control treatment). On the other hand, plant RWC and MSI indices were substantially elevated by 8.3%, 10.6%, and 14.9% and 12.5%, 13.9%, and 17.8%, respectively, in sweet potato cultivated in Cd^2+^-contaminated saline soil in response to MLE, EMs, or MLE plus EMs over the control (Table 3). Similarly, Cd^2+^-contaminated saline soil exhibited negative impacts and significantly diminished the photosynthetic pigment contents (i.e., chl “a”, chl “b”, total chlorophyll, and carotenoids) of sweet potato plants. However, the individual or integrative application of MLE and EMs alleviated the salt and Cd^2+^-induced damages to the photosynthetic pigments. Sweet potato plants treated with MLE or EMs showed higher values of chl “a” by (86.6 and 35.1%), chl “b” (145.4 and 94.1%), total chlorophyll (119.0 and 67.6%), and carotenoids (118.2 and 81.8%), respectively, as compared to the untreated plants. Moreover, exogenous MLE plus soil application of EMs further increased the photosynthetic pigment contents of stressed sweet potato plants. The highest results of chl “a” (2.22), chl “b” (3.81), total chlorophyll (6.03), and carotenoids (0.26) as an average of the two seasons were measured for treated sweet potato plants with MLE + EMs. Interestingly, the MLE + EMs treatment exhibited non-significant differences with the MLE treatments for carotenoids.

**Table 3:** Influence of *Moringa* leaf extract (MLE) and effective microorganisms (EMs) on membrane stability index (MSI), and relative water content (RWC), as well as chlorophyll “a” (Chl a), chlorophyll “b” (Chl b), total chlorophyll, and carotenoid contents of sweet potato grown in Cd2 + -contaminated saline soil in the 2020 and 2021 seasons (as average)

Factors	RWC (%)	MSI (%)	Chl a (mg g^−1^ FW)	Chl b (mg g^−1^ FW)	Total Chl (mg g^−1^ FW)	Carotenoids (mg g^−1^ FW)
Growing season (GS)						
GSI	75.2 ± 0.62a	66 ± 1.4b	1.8 ± 0.14a	2.7 ± 0.22a	4.2 ± 0.42a	0.21 ± 0.10a
GSII	77.6 ± 0.71a	74 ± 1.2a	1.4 ± 0.12b	2.5 ± 0.24b	4.0 ± 0.33a	0.20 ± 0.09a
Treatments						
Control	70.48 ± 0.51c	62.81 ± 1.69c	0.97 ± 0.13d	1.19 ± 0.16c	2.16 ± 0.26d	0.11 ± 0.08c
MLE	76.33 ± 0.33b	70.67 ± 1.88b	1.81 ± 0.13b	2.92 ± 0.36b	4.73 ± 0.38b	0.24 ± 0.10a
EMs	77.98 ± 0.94b	71.56 ± 1.65b	1.31 ± 0.18c	2.31 ± 0.29b	3.62 ± 0.47c	0.20 ± 0.10b
MLE + EMs	81.00 ± 0.58a	74.00 ± 1.58a	2.22 ± 0.21a	3.81 ± 0.31a	6.03 ± 0.51a	0.26 ± 0.11a
*p*-value						
GS	0.084	0.032	0.035	0.143	0.131	0.072
Treatment	< 0.001	0.003	< 0.001	< 0.001	< 0.001	0.045
CV%	1.40	1.70	9.20	13.70	11.10	16.6

The various lower-case letters on the bar distinguished the treatments, corresponding to Fisher’s test at *p* ≤ 0.05

### Osmolyte accumulation

**Fig. 1:**
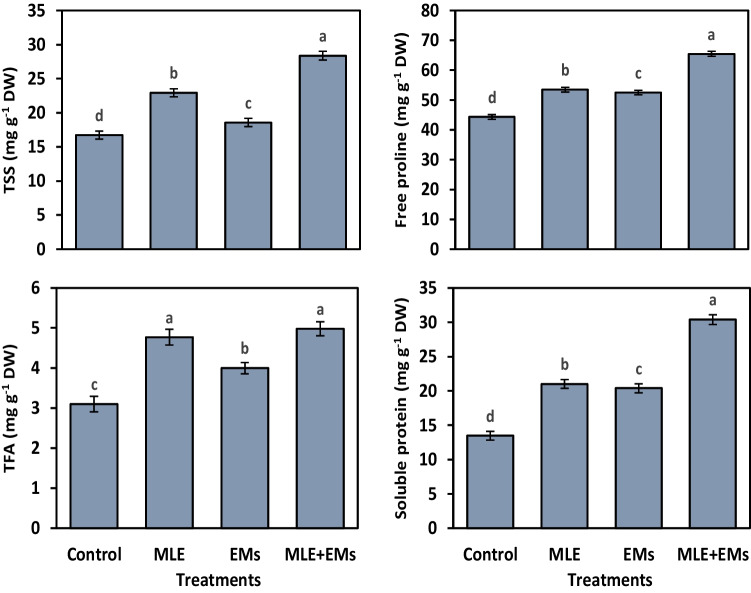
Influence of *Moringa* leaf extract (MLE) and effective microorganisms (EMs) on osmolytes (i.e., free proline, total free amino acids—TFA, total soluble sugars—TSS, and soluble proteins) content of sweet potato plants grown in Cd2 + -contaminated saline soil in the 2020 and 2021 seasons (as average). The various lower-case letters on the bar distinguished the treatments, corresponding to Fisher’s test at *p* ≤ 0.05

The data in Fig. 1 represented the accumulation of osmolytes (i.e., TSS, free proline, TFA, and soluble proteins) in sweet potato grown under Cd^2+^-contaminated saline soil in response to applied plant biostimulants. The accumulation of these osmoprotectants increased significantly in correspondence with the individual and co-use of MLE and EMs. The application of EMs to Cd^2+^-contaminated saline soil generated a significant increase in contents of TSS (by 11.0%), free proline (by 18.4%), TFA (by 53.9%), and soluble proteins (by 51.4%) in leaves of sweet potato plants in comparison to the control. In addition, applying MLE as a foliar spray on stressed sweet potato plants caused a significant increase in the contents of the osmo-regulators mentioned above by 37.1, 20.6, 60.6, and 55.9%, respectively, in contrast to the control. Combined application of MLE and EMs resulted in a substantial increase in concentrations of TSS by 69.6%, free proline by 47.7%, TFA by 29.0%, and soluble proteins by 125.7% compared with untreated plants. Meanwhile, the difference in free proline content due to the single addition of MLE or in combination with EMs was insignificant. Hence, in correspondence with used agro-management practices, these substances’ accumulation followed the subsequent descending order: MLE + EMs > MLE > EMs.

### Antioxidative compounds

**Fig. 2:**
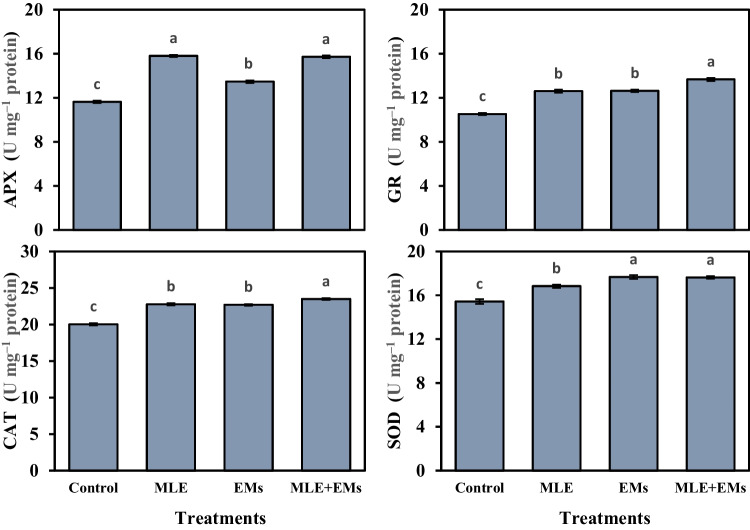
Influence of *Moringa* leaf extract (MLE) and effective microorganisms (EMs) on the activity of glutathione reductase (GR), superoxide dismutase (SOD), catalase (CAT), and ascorbate peroxidase (APX) of sweet potato plants grown in Cd2 + -contaminated saline soil in the 2020 and 2021 seasons (as average). The various lower-case letters on the bar distinguished the treatments, corresponding to Fisher’s test at *p* ≤ 0.05

**Fig. 3:**
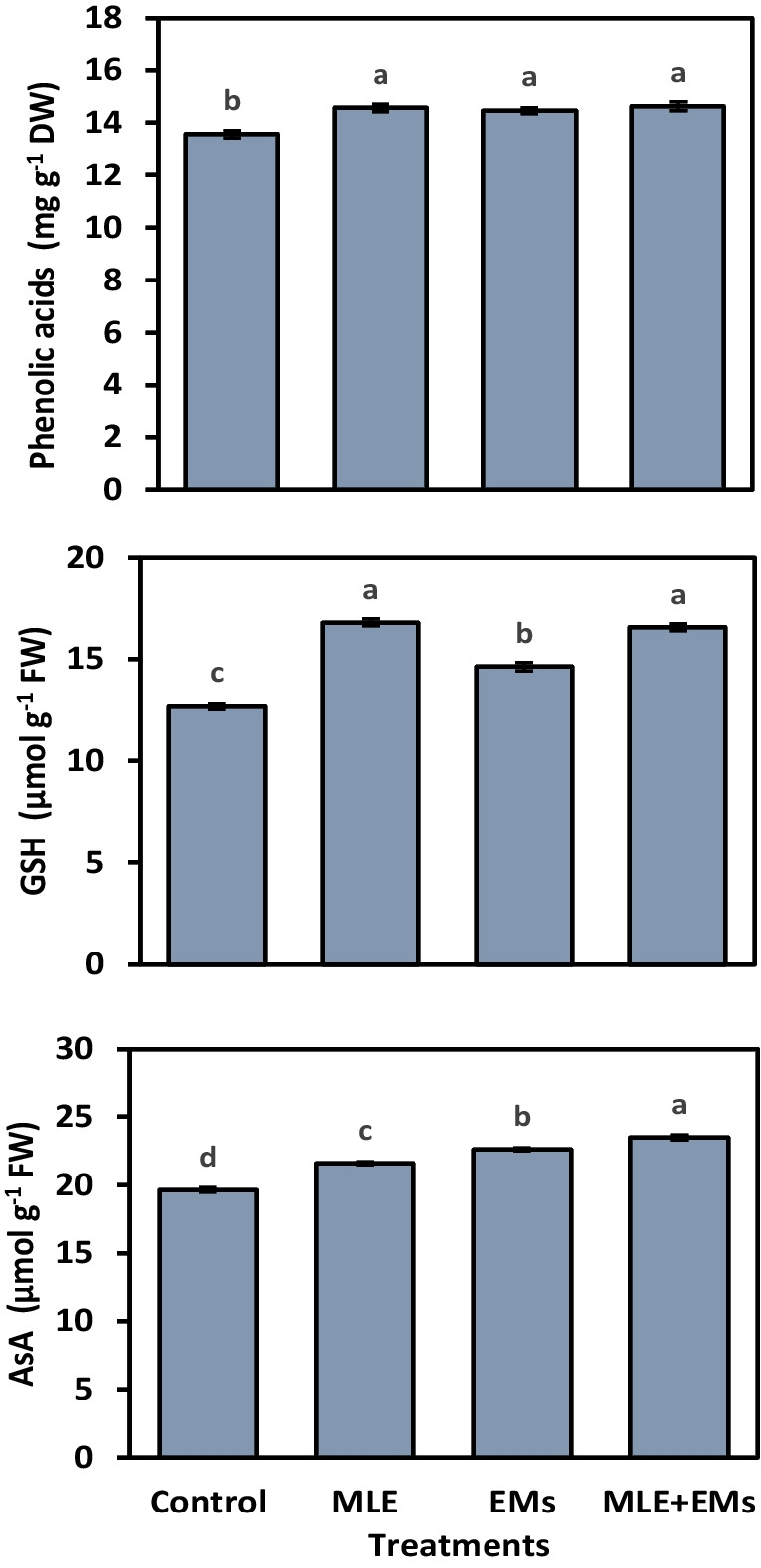
Influence of *Moringa* leaf extract (MLE) and effective microorganisms (EMs) on phenolic acids, ascorbic acid (AsA), and glutathione (GSH) contents of sweet potato plants grown in Cd2 + -contaminated saline soil in the 2020 and 2021 seasons (as average). The various lower-case letters on the bar distinguished the treatments, corresponding to Fisher’s test at *p* ≤ 0.05

Our results indicated that the minimum values of ascorbate peroxidase (APX), glutathione reductase (GR), catalase (CAT), and superoxide dismutase (SOD) at 11.63, 10.53, 20.03, 15.43 U mg^‒1^ protein, respectively, were observed for non-treated sweet potato plants. Interestingly, the activity of the enzymatic antioxidants APX, GR, CAT, and SOD was upregulated in sweet potato plants subjected to Cd^2+^-contaminated saline soil by the application of MLE, EMs, or MLE plus EMs (Fig. 2); they elevated the APX by 35.9, 15.8, and 35.3%, the GR by 19.7, 19.9, and 29.8%, the CAT by 13.7, 13.3, and 17.3%, and the SOD by 9.1, 14.5, and 14.3%, respectively, in comparison with the control. In Cd^2+^-contaminated saline soil, the single or co-application of MLE and EMs accumulated higher phenolic acids, GSH, and AsA in sweet potato leaves (Fig. 3). These treatments raised phenolic acids (6.6–7.8%), GSH (5.5–7.0%), and AsA (9.9–19.6%) compared to the control plants. Single or combined application of MLE and EMs recorded similar values for phenolic acids and GSH while noting the superiority of MLE + EMs treatment in AsA.

### Nutrient acquisition

**Fig. 4:**
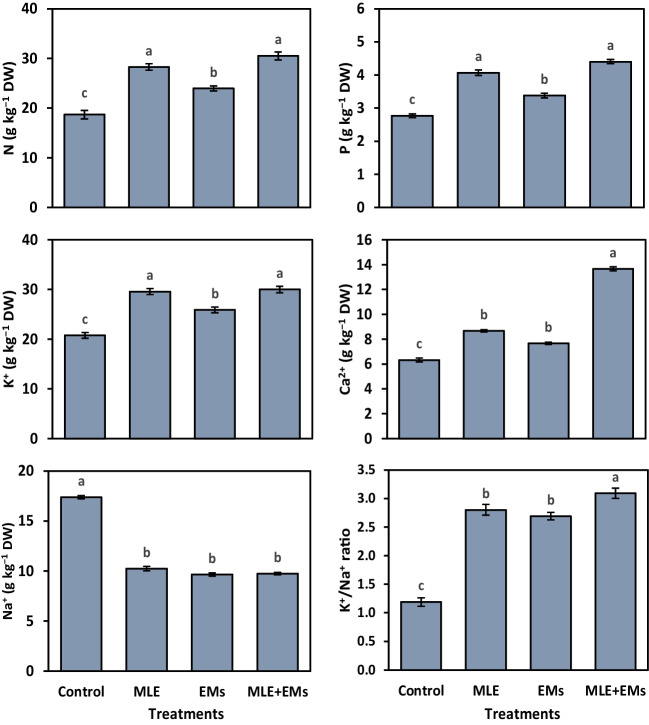
Influence of *Moringa* leaf extract (MLE) and effective microorganisms (EMs) on leaf nutrient concentrations of sweet potato grown in Cd2 + -contaminated saline soil in the 2020 and 2021 seasons (as average). The various lower-case letters on the bar distinguished the treatments, corresponding to Fisher’s test at *p* ≤ 0.05

Results listed in Fig. 4 showed significant improvements in the nutrient acquisition of sweet potato plants cultivated in Cd^2+^-contaminated salty soil due to the MLE and/or EMs treatments. The uptake of nutrient elements (i.e., N, P, K^+^, Ca^2^) and the K^+^/Na^+^ ratio by sweet potato plants grown in control plots was substantially reduced compared with MLE and EMs amended plants. Unlike the nutrients mentioned above, the levels of Na^+^ were elevated in control plots in sweet potato leaves. Nevertheless, foliar-applied MLE or EMs triggered a notable improvement in N, P, K^+^, Ca^2+^, and K^+^/Na^+^ ratio contents by 51.3 or 28.2%, 46.9 or 22.0%, 42.5 or 24.8%, 37.0 or 21.2%, and 135.0 or 126.1%, respectively, while decreasing the Na^+^ content by 41.0 or 44.4% in sweet potato plants relative to control. Interestingly, the integrative application of MLE and EMs magnified the macronutrient concentration, bypassing individual influences. They caused N to rise by 63.2%, P to rise by 58.8%, K^+^ to rise by 44.5%, Ca^2+^ to rise by 116.0%, and K^+^/Na^+^ ratio to rise by 159.7%, and Na^+^ to decrease by 43.9% when compared to control.

### Roots and leaves cadmium (Cd^2+^) content

**Fig. 5:**
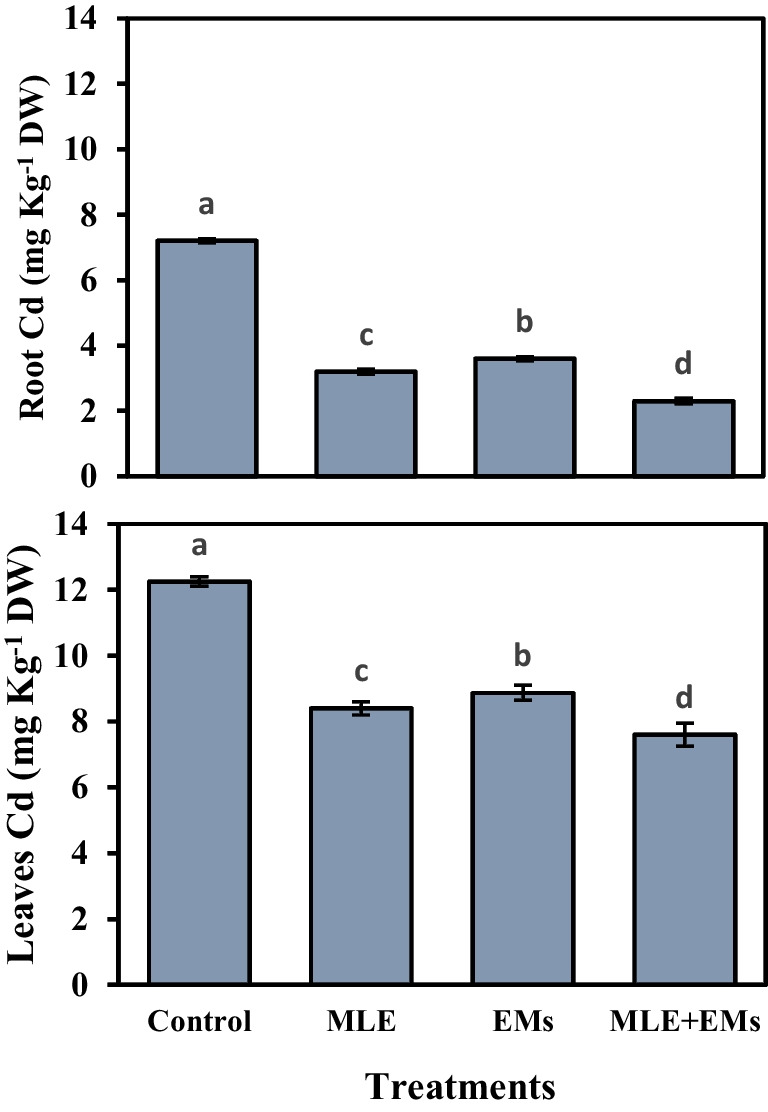
Influence of *Moringa* leaf extract (MLE) and effective microorganisms (EMs) on the Cd2 + content in roots and leaves of sweet potato grown in Cd2 + -contaminated saline soil in the 2020 and 2021 seasons (as average). The various lower-case letters on the bar distinguished the treatments, corresponding to Fisher’s test at *p* ≤ 0.05

Sweet potato plants grown in saline soil contaminated with Cd^2+^ exhibited higher accumulation of Cd^2+^ in the roots and leaves (Fig. 5). Nonetheless, foliar-applied MLE, soil application with EMs, or their integrative application significantly reduced Cd^2+^ content in the roots and leaves of sweet potato plants transplanted in Cd^2+^-contaminated saline soil (Fig. 5). The best results were associated with MLE + EMs treatment, which maintained the accumulation of Cd^2+^ ions at their lowest levels in the roots (68.1%) and leaves (38.0%) compared to the control. Also, the application of MLE or EMs induced a significant reduction of Cd^2+^ content in the roots by 55.6 or 50.0% and in the leaves by 31.4 or 27.6%, respectively, compared with the control.

### Yield and yield-related components

Sweet potato plants grown in Cd^2+^-contaminated saline soil produced the lowest tubers’ number per plant, tuber weight, tubers’ weight per plant, and tuber yield (Table 4). Nonetheless, Cd^2+^- and salt-stressed *Ipomoea batatas* plants exhibited an increment in the yield-related components when treated with MLE, EMs, or MLE plus EMs. The increments of the aforementioned yield-related characters amounted to 35.3 or 34.6%, 59.4 or 35.7%, 19.7 or 17.9%, and 59.5 or 35.7%, respectively, due to exogenous MLE or soil application of EMs against control. Meanwhile, the integrative application of MLE and EMs generated further increases in tuber number per plant by 76.4%, tuber weight by 44.7%, tubers’ weight per plant by 73.0%, and tuber yield by 73.1% (Table 4).

**Table 4:** Influence of *Moringa* leaf extract (MLE) and effective microorganisms (EMs) on the yield-related components of sweet potato grown in Cd2 + -contaminated saline soil in the 2020 and 2021 seasons (as average)

Factors	Tubers no. plant^−1^	Tubers weight plant^−1^ (g)	Tuber weight (g)	Tuber yield (*t* ha^−1^)	WUE (kg m^−3^)
Growing season (GS)					
GSI	7.7 ± 1.3a	1101 ± 20.1a	141.2 ± 3.2a	27.1 ± 1.2a	3.3 ± 0.24a
GSII	7.9 ± 1.4a	1054 ± 17.5b	138.2 ± 4.1a	26.4 ± 0.98b	3.0 ± 0.16b
Treatments					
Control	5.67 ± 0.33c	759 ± 19.4d	114.4 ± 3.1c	18.97 ± 0.48d	2.28 ± 0.08d
MLE	7.67 ± 1.2b	1210 ± 10.0b	136.9 ± 4.2b	30.25 ± 0.25b	3.63 ± 0.13b
EMs	7.63 ± 0.33b	1030 ± 17.3c	134.9 ± 4.5b	25.75 ± 0.43c	3.09 ± 0.15c
MLE + EMs	10.00 ± 1.2a	1313 ± 20.2a	165.5 ± 5.9a	32.83 ± 0.44a	3.94 ± 0.11a
*p*-value					
GS	0.201	< 0.001	0.152	0.072	0.024
Treatment	< 0.001	< 0.001	0.048	< 0.001	< 0.001
CV%	6.50	2.50	18.20	2.50	2.50

The various lower-case letters on the bar distinguished the treatments, corresponding to Fisher’s test at *p* ≤ 0.05

### Relationships

**Fig. 6:**
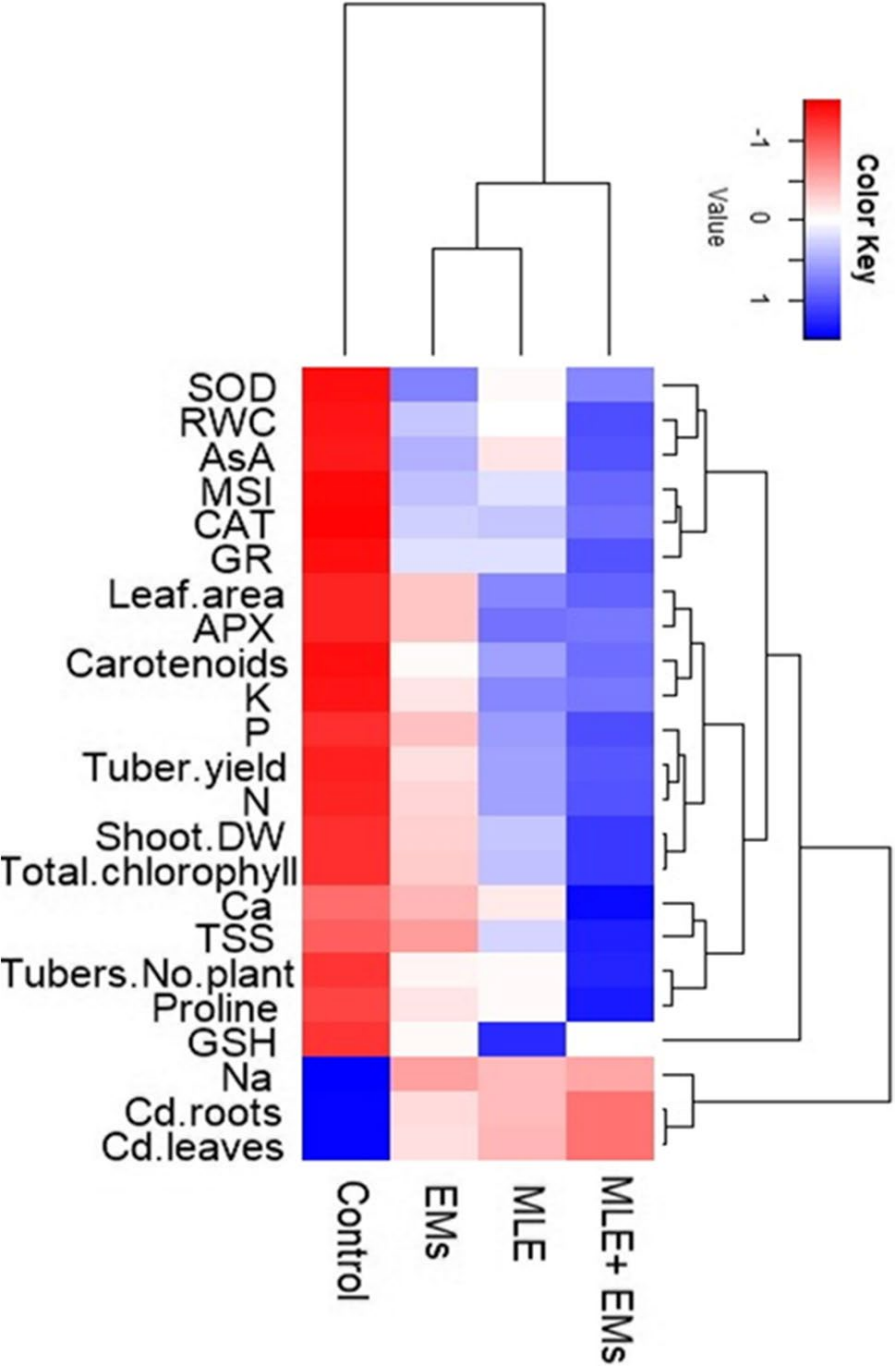
The heat map diagram displays hierarchical clustering analysis among the application of *Moringa* leaf extract (MLE) and/or effective microorganisms (EMs) and different studied parameters of sweet potato plants grown Cd2 + -contaminated saline soil. SOD, superoxide dismutase; RWC, relative water content; AsA, ascorbic acid; MSI, membrane stability index; CAT, catalase; APX, ascorbate peroxidase; GR, glutathione reductase; Leaf.area, leaf area plant^−1^; K, potassium; P, phosphorus; N, nitrogen; Ca, calcium; Na, sodium; Shoot DW, shoot dry weight; TSS, total soluble sugars; Tubers. no. plant, tubers number plant-1; GSH, glutathione; Cd. root and Cd. Leaves, roots and leaves Cd2 + content, respectively

**Fig. 7:**
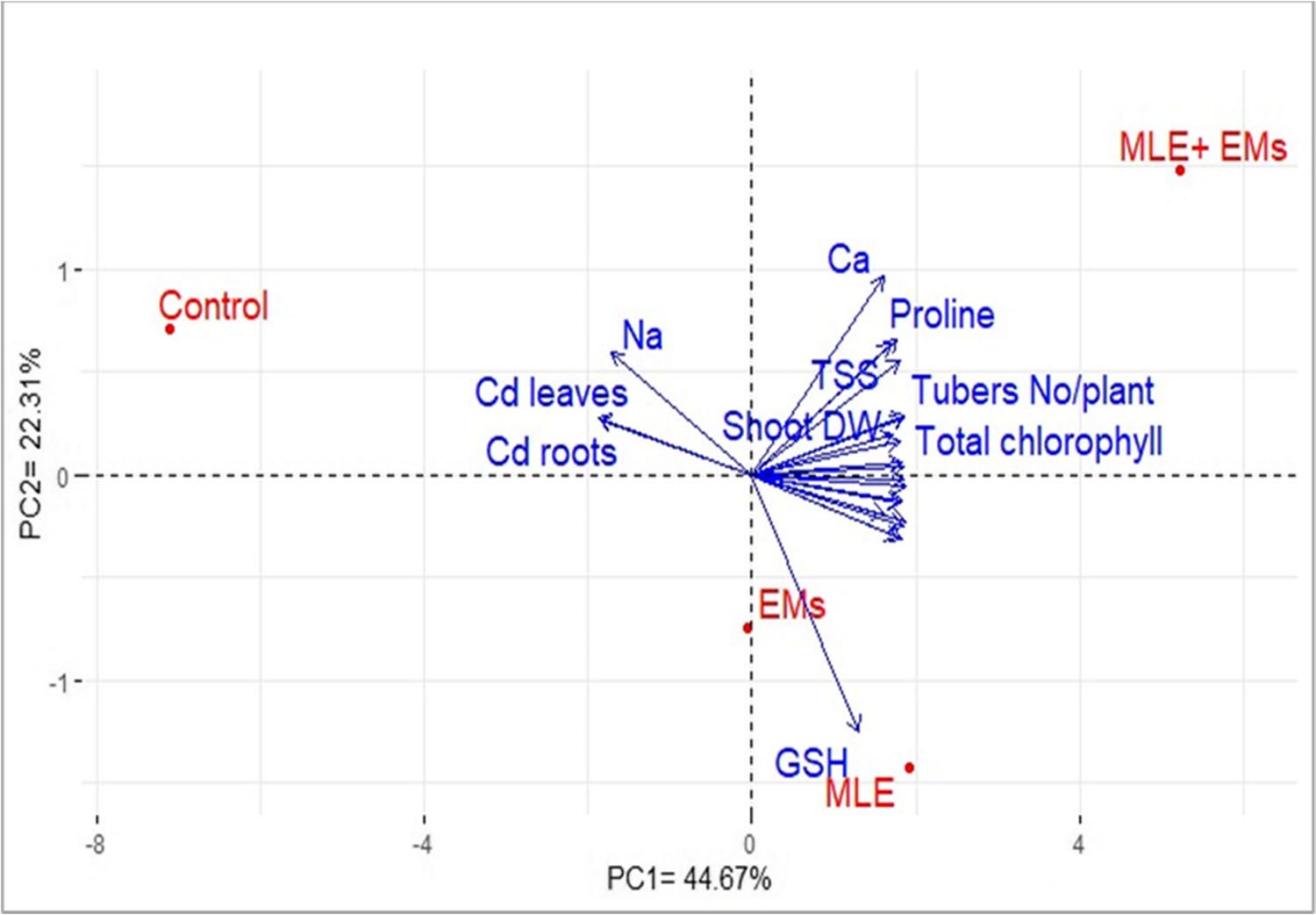
Biplot of the principal component analysis (PCA) illustrating the relationship among the effects of *Moringa* leaf extract (MLE) and/or effective microorganisms (EMs) on different assessed parameters of sweet potato plants grown in Cd2 + -contaminated saline soil. Ca, calcium; *Na*, sodium; proline, free proline; TSS, total soluble sugars; GSH, glutathione; Shoot DW, shoot dry weight; Cd root and Cd leaves, roots and leaves Cd2 + content, respectively

The hierarchical analysis presented in a heat map graph shows the relation between the single or combined application of MLE and EMs on the studied parameters (Fig. 6), divided into two major sets (control and application of EMs, MLE, or MLE and EMs). The hierarchical analysis exhibited that the co-application MLE and EMs separated in a sub-set and performed well from the individual application of MLE or EMs. These results indicated that the foliar spraying MLE and soil application of EMs to sweet potato plants grown in Cd^2+^-contaminated saline soil have a significant role in enhancing the growth, yield, and defense machinery of the plant while reducing the Na^+^ and Cd^2+^ accumulation in sweet potato tissues. The impact of MLE and/or EMS on agronomic, physiological, and biochemical parameters under Cd^2+^ and salt stress conditions was investigated using the principal component analysis (PCA) (Fig. 7). The variability of the first and second components represents about 66.98% (PC1 = 22.31 and PC2 = 44.67%) (Fig. 7). Under Cd^2+^-contaminated saline soil, the PCA showed a positive correlation of the integrative application of MLE and EMs for growth, tuber yield, photosynthetic pigments, osmolyte accumulation, and antioxidant capacity. The analysis also revealed a positive correlation between Cd^2+^-contaminated saline soil and Na^+^, Cd^2+^ accumulation in sweet potato plants. Also, under Cd^2+^ and salt stress, PCA exhibited a clear positive correlation between GSH and the individual effect of MLE or EMs.

## Discussion

Heavy metal-contaminated salty soil seriously threatens the sustainability of vegetable crop production (Abd El-Mageed et al. 2022a, b). As a result, a new challenge arises in increasing sweet potato tolerance, reducing the unfavorable conditions brought on by the co-occurrence of Cd^2+^ accumulation and salinity in the soil, and promoting their long-term sustainability. Therefore, the application of biostimulants for plants has been suggested to support them to withstand Cd^2+^ and salt stress. Furthermore, since the co-application of MLE and EMs has never been done, it is crucial to comprehend further the mechanism of action of exogenous MLE and soil application of EMs on sweet potato under Cd^2+^ and salt stress.

Long-term exposure to saline soil contaminated with heavy metals perturbed several metabolic processes, including cell division, elongation, cell cycle arrest, and a high transpiration rate because of a high energy requirement (Desoky et al. 2019b). As a result, sweet potato plant growth was negatively impacted (Table 2), and tuber yield was decreased (Table 4). Plant growth suppression caused by Cd^2+^-contaminated saline soil-mediated stress might be attributed to ROS generation, photo-inhibition, reduced photosynthetic pigment contents, and plant water status. Also, increased Cd^2+^ and Na^+^ acquisition in sweet potato plants may cause a nutritional imbalance and ion toxicity, leading to symptoms of nutritional deficiency and growth restrictions (Isayenkov and Maathuis 2019; dos Santos et al. 2022). However, exogenous MLE combined with soil-applied EMs enhanced stressed sweet potato plants’ growth characteristics and physiological and biochemical attributes. Protein-containing MLE is necessary for protoplasm formation, vitamin C, and essential nutrients like Ca^2+^, Mg^2+^, and K^+^ that are involved in several natural antioxidant compounds such as flavonoids, ascorbic acid, phenolic acids, and carotenoids which in turn reflect its vital role as an excellent growth biostimulator (Talaat 2019; Abd El-Mageed et al. 2022a, b).

Furthermore, the application of auxins and cytokinins (especially zeatin) and gibberellins-containing MLE has a growth-promoting effect that improves cell division and enlargement and enhances the biosynthesis of chlorophyll molecules. Similar results showed MLE’s positive effect on wheat plants’ growth (Ahmed et al. 2021) and maize (Pervez et al. 2017). Similarly, improved growth traits and tuber yield in EMs-treated sweet potato plants could be attributed to synthesizing some useful bioactive material for plants, such as amino acids, lactic acid, vitamins, sugars, enzymes, and hormones that trigger cell division (Hu and Qi 2013). Furthermore, EMs stimulate the production of several hormones such as abscisic acid, cytokinin, and auxin; the accumulation of EPSs and enzymes (i.e., 1-aminocyclopropane-1-carboxylate deaminase (ACC deaminase); the synthesis of osmoregulatory substances (Bittencourt et al. 2023). These actions collectively induce the osmoregulation process, which may has a beneficial effect on sweet potato tolerance to saline and Cd^2+^ stress (Rady et al. 2023).

In response to Cd^2+^ or/and salt stress, plants accumulate extra osmoregulators such as soluble sugars, free proline, free amino acids, and soluble proteins to contribute to stress tolerance (Abdelkhalik et al. 2019; Semida et al. 2021a). These solutes help plants to adjust their osmotic environment, maintain turgor pressure, allow more nutrients and water uptake, inhibit free radicals’ activity, and safeguard their thylakoid membranes (Semida et al. 2020; Abdou et al. 2022). The single use of MLE or coupled addition with EMs generated further accumulation of soluble sugars, free proline, free amino acids, and soluble protein in Cd^2+^ and salt-stressed sweet potato plants (Fig. 1). Moreover, the greatest favorable results were assigned to the co-application of MLE and EMs. This favorable effect of MLE on stressed sweet potato plants could be associated with high content of osmolytes, phytohormones, and mineral ions (Zulfiqar et al. 2020; Khan et al. 2022). These beneficial MLE constituents were also identified in the MLE analysis conducted for this study (Table S1), which may have contributed to the accumulation of these osmoprotectants in MLE-treated plants. Exogenously applied MLE as an activator for free proline accumulation was observed in other crops, such as beans (Rady and Mohamed 2015) and potatoes (El-Mohamed et al. 2016).

In addition, when plants are exposed to unfavorable conditions, they activate the synthesis of antioxidants to mitigate the detrimental effects of oxidative stress caused by free radicals (Abd El-Mageed et al. 2022a, b; Selem et al. 2022). The present study showed significant increases in the activity of antioxidant enzymes (i.e., GR, APX, CAT, and SOD; Fig. 2) and the content of non-enzymatic antioxidants (i.e., phenolic acids, GSH, and AsA; Fig. 3). This improvement in GSH, AsA, GR, CAT, SOD, and APX levels is an ameliorative mechanism by which sweet potato plants could resist the oxidative stress generated via Cd^2+^ and salinity exposure. Additionally, these findings exhibited that MLE or/and EMs may combat oxidative stress by improving the AsA-GSH cycle and upregulating the enzymatic antioxidants. This significant improvement could be attributed to the effective role of MLE as a good source of amino acids, phenolic acids, nutrients, soluble sugars, and antioxidants like ascorbate and free proline (Table S1), which subsequently enhanced the accumulation of these antioxidants and enabled sweet potato plants to tolerate Cd^2+^ and salinity stress. Besides, its considerable levels of carotenoids (α, β carotene, lutein, and xanthin) have potent antioxidant properties (Irshad et al. 2022; Khan et al. 2022; Martínez-ispizua et al. 2022a). A similar finding was observed by Zulfiqar et al. (2020) and Khalofah et al. (2020), who reported that MLE application caused a considerable increase in GR, SOD, and APX antioxidants in common bean plants. Inculcating plants with EMs might enhance the synthesis of EPSs and beneficial enzymes, such as ACC deaminase and phytohormones (i.e., cytokinin, auxin, and abscisic acid), which in turn may lessen the harm caused by environmental stress (Bittencourt et al. 2023). This positive impact of EMs on the plant antioxidant defense system was also represented by Iriti et al. (2019) and Abd El-Mageed et al. (2022a, b). Hence, activating antioxidative enzymes, protecting the plants from oxidative damage via regulating some metabolic processes, including cell division, biosynthesis of proteins, the activity of enzymes, phytohormones, and gene expression, protecting cells from H_2_O_2_ toxicity, and maintaining membranes from oxidation and organic peroxides (Patel and Parida 2021; Martínez-ispizua et al. 2022b).

In the present study, planting sweet potatoes in Cd^2+^-contaminated saline soil led to a higher accumulation of Cd^2+^ and Na^+^ in plant tissue (Figs. 4 and 5), resulting in a reduction in nutrient acquisition (Fig. 4), plant water status, membrane integrity, and photosynthetic pigment contents (Table 3), as well as growth (Table 2), and tuber yield (Table 4). However, applying MLE or/and EMs ameliorated the Cd^2+^-contaminated saline soil-induced damage to sweet potatoes. The Cd^2+^ and salinity stress mediated disturbances in the ion homeostasis of sweet potato, given that they raised leaf Cd^2+^ and Na^+^ and lowered N, P, K^+^, Ca^2+^, and the K^+^/Na^+^ ratio (Fig. 4). This could be due to Na^+^ and Cd^2+^ influx that may disturb water uptake and interfere with the same N, P, K + , Ca^2+^, Mn, and Mg transporters, restricting their absorption (Sales and Martins 2019; Sitohy et al. 2020; Haider et al. 2021). Nonetheless, externally applied MLE or MLE plus EMs-treated soil alleviated the cumbersome influences of toxic Na^+^ and Cd^2+^ by reducing their content in the tissues and induced a considerable increase in N, P, K^+^, and Ca^2+^ acquisition, increasing the K^+^/Na^+^ ratio. As a key component of salt tolerance, maintaining high K^+^/Na^+^ ratios in tissues and, consequently, high cytosolic K^+^/Na^+^ ratios has been used to explain how Na^+^ uptake and transport are regulated in plants under salt stress (Assaha et al. 2017). Our study demonstrated a higher accumulation of Cd^2+^ in the sweet potato leaves than in the roots. In harmony with our data, Baldantoni et al. (2016) reported that the Cd^2+^ content in lettuce and endive leaves raised to 9 mg.kg^−1^, fourfold higher than in roots, and up to 30-fold higher than the Cd^2+^ content in the studied soil.

Foliar-applied MLE and soil-treated EMs alleviated the harmful effect of excessive Cd^2+^ and Na^+^ in root media by reducing its uptake and considerably increased the acquisition of essential macronutrients. Hence, improving the nutritional status of stressed sweet potato plants by lowering the absorption of Cd^2+^ and Na^+^ ions and raising the acquisition of N, P, K^+^, and Ca^2+^ using MLE and EMs biostimulants could be a vital strategy to counteract Cd^2+^ and salt stress. The nutritional profile of MLE-treated sweet potato plants was improved because MLE is a natural product abundant in vital elements, including Ca^2+^, Mg^2+^, K^+^, P, Fe^2+^, Mn^2+^, Cu^2+^, and Zn^2+^ (Muthuraja and Muthukumar 2022). The effective function that EMs play in preserving greater membrane integrity that consequently controls the membranes’ ability to selectively take in and transport ions (Munir et al. 2022) may be the cause of improved nutrient uptake with EMs’ application. Inoculating the soil with EMs accelerates the organic matter decomposition, which boosts nutrient release and solubility and directly impacts plant ion uptake (Soares et al. 2021; Abdelkhalik et al. 2023).

Furthermore, increased protein production in response to using MLE alone or combined with EMs on sweet potato plants might be correlated with increased content of inorganic ions such as N, P, K^+^, and Ca^2+^, and a higher K^+^/Na^+^ ratio. Plant nutritional status ameliorates the disruptive effect of osmotic stress on various enzymes, including protein biosynthesis (Talaat 2019). EMs can accumulate osmolytes, which synergistically can act with osmoprotectants produced by plants, such as free proline and total soluble sugars (Ozturk et al. 2021). Moreover, plants treated with EMs can produce exopolysaccharides (EPSs), enabling plants to have a more substantial accumulation of sugars, free proline, TFA, and soluble protein content (Khan and Bano 2019). As a result, these beneficial bacteria cause the manufacture of these osmolytes in plant cells, which protects cells from oxidative damage and increases tolerance to Cd^2+^ or/and salt stress (Rady et al. 2021; Ahmed et al. 2022).

High levels of Cd^2+^ and salt in the soil lower their osmotic potential and impose a decrease in leaf water potential, causing osmotic stress (Rucińska-Sobkowiak 2016) and a reduction of RWC as stated in the present study (Table 3). Thereafter, these salts accumulated in the plant tissues, inducing oxidative damage (Abd El-Mageed et al. 2020) and causing damage to the cellular membrane (MSI; Table 3). However, the applied bio-elicitors, MLE and/or EMs, resulted in higher RWC and MSI than Cd^2+^ and salt-stressed sweet potato plants, indicating that the osmotic and ionic stress was lower in MLE and/or EMs-treated plants (Talaat et al. 2015). The beneficial effect of MLE and/or EMs on tissue RWC could be attributed to their vital role in regulating the osmotic potential inside plant cells via increasing the content of TSS, TFA, free proline (Fig. 1), and inorganic ions such as K^+^ (Fig. 4). Also, stimulated ion acquisition (Fig. 4) in response to MLE and/or EMs application decreases the toxic effects of Na^+^ and Cd^2+^, improving the stability of membranes in stressed sweet potato plants. The enhancements in MSI generated by EMs application may be attributed to EMs’ beneficial effects on plant defense mechanisms, such as free proline (Fig. 1), enzymatic and non-enzymatic antioxidants (Figs. 2 and 3), and carotenoid accumulation (Table 3), which is crucial for maintaining cell membrane stability by preventing ROS accumulation (Talaat et al. 2015; Abd El-Mageed et al. 2022a, b). MLE is rich in Ca^2+^ ions required for structural roles in the cell membranes, improving Ca^2+^ status in plant tissues, which minimizes ion leakage and enhances membrane stability for MLE-treated plants (Farhat et al. 2018; Al-Taisan et al. 2022).

The Cd^2+^ and salt stress exacerbate lower physiologically active water in tissues, ROS production, and nutrient acquisition, thus activating the chlorophyllase enzyme and chloroplast deterioration, inhibiting photosynthetic pigment biosynthesis (Rasafi et al. 2022). However, the unfavorable photosynthetic pigments from salty soil contaminated with Cd^2+^ were alleviated by the co-application of MLE and EMs, which may be related to their positive roles in increased nutrient acquisition, osmoprotectants, and antioxidants. These results are harmonized with those demonstrated by Talaat et al. (2015) and Kalaji et al. (2016). The presence of nutrients, osmolytes, cytokinins (Zeatin), and gibberellins in MLE (Table S1) stimulates the mechanisms involved in chlorophyll and carotenoids biosynthesis, inhibits photosynthetic pigment degradation and premature leaf senescence, and maintains leaf greenish for longer periods (Desoky et al. 2019b). Applying foliar spraying with MLE significantly mitigated the observed enhancements in chlorophyll levels in wheat plants subjected to drought stress (Fozia et al. 2022). Furthermore, as a biostimulator, MLE is rich in different nutritional elements, especially Mg, a constituent of chlorophyll molecules. Also, the EMs’ application may stimulate chlorophyll biosynthesis due to its effective role in improving the absorption and translocation of essential ions such as N, P, K^+^, and Ca^2+^.

The present study found that sweet potatoes grown in saline soil contaminated with Cd^2+^ had a larger buildup of Cd^2+^ in their roots and leaves (Fig. 5). Interestingly, the integrative application of MLE and EMs substantially reduced Cd^2+^ accumulation in different sweet potato parts grown in Cd^2+^-contaminated salty soil (Fig. 5). Therefore, applying biostimulants (i.e., MLE and EMs) is considered an effective strategy for Cd^2+^-phytoremediation when applied as foliar and soil applications. EMs may be able to adsorb Cd^2+^ ions (Treesubsuntorn et al. 2018; Matos et al. 2019), thus reducing their transportation into sweet potato plants. Furthermore, increased availability of Cd^2+^-divalent cations such as Ca^2+^ and Mg^2+^ may create competition with Cd^2+^, decreasing Cd^2+^ uptake in rice plants cultivated in Cd^2+^-contaminated soil (Treesubsuntorn et al. 2018). Cd^2+^ reduction in sweet potato by exogenous MLE might be a result of the production of enzymatic (Fig. 2) and non-enzymatic antioxidants (Fig. 3) and increased nutrient acquisition to counteract Cd^2+^ sequestration in plants (Khalofah et al. 2020, Abalaka et al. 2022).

The positive effects of used MLE and/or EMs on sweet potato plants’ growth and physiological and biochemical responses are reasonable for improved water use efficiency, yield, and related parameters (Table 4). In another study by Abd El-Mageed et al. (2022a, b), saline soil-applied EMs increased sweet potato plants’ average tuber weight, number, and yield.

Our study demonstrated the possibility of foliar application with MLE and/or soil application of EMs to improve sweet potato growth and productivity in Cd^2+^-contaminated saline soil. Greater effectiveness was demonstrated in decreasing stress brought on by Cd^2+^ and salinity when MLE and EMs were applied together rather than separately. Moreover, the present results may have practical importance as they highlight the potential of both EM and MLE as successful plant biostimulants for future use in reducing oxidative damage and promoting sustainable crop production in arid and semiarid areas.

## Conclusion

The results showed that the integrative application of *Moringa* leaf extract and effective microorganisms can effectively enhance sweet potato tolerance to salinity and cadmium stress. Stress tolerance in sweet potato is a result of increased nutrient acquisition, osmolytes accumulation, and antioxidants in sweet potato plants compared with control. Moreover, *Moringa* leaf extract and effective microorganisms beneficially affected stressed plants by reducing the cadmium and sodium uptake in sweet potato tissues. Therefore, using these biostimulants may help plants thrive in challenging environments brought on by salt and cadmium stressors. The photosynthetic pigments, tissue water status, and membrane stability were improved in plants treated with *Moringa* leaf extract and effective microorganisms; subsequently, the growth and tuber yield of sweet potato plants were promoted. Therefore, using *Moringa* leaf extract and effective microorganisms to increase the productivity of sweet potato plants cultivated in cadmium-contaminated saline soils may be a promising sustainable strategy for the future.

### Supplementary Information

Below is the link to the electronic supplementary material.

Supplementary file1 (DOC 39 KB)

## Data Availability

The data behind this article will be shared with the appropriate authors upon reasonable request.
