# Supplementary material for: Enhancing sweet potato (Ipomoea batatas) resilience grown in cadmium-contaminated saline soil: a synergistic approach using Moringa leaf extract and effective microorganisms application
Source: Environ Sci Pollut Res Int. 2024 Apr 23;31(22):32464–79. doi: 10.1007/s11356-024-33295-w (PMC11133164; doi:10.1007/s11356-024-33295-w)
Supplement: Supplementary file 1 — Supplementary file1 (DOC 39 KB) [file 11356_2024_33295_MOESM1_ESM.doc]

S1. Some chemical constituents of Moringa oleifera leaf extract (on dry weight basis)

| Component | Value (mg g^−1^ DW) |
| --- | --- |
| Amino acids | 124.7 |
| Proline | 26.09 |
| Total soluble sugars | 151.4 |
| Ash | 111.3 |
| Magnesium | 6.035 |
| Calcium | 8.756 |
| Potassium | 27.68 |
| Phosphorus | 6.122 |
| Sodium | 0.674 |
| Manganese | 0.966 |
| Zinc | 0.453 |
| Copper | 0.208 |
| Soluble phenols | 2.252 |
| Total carotenoids | 2.243 |
| Total chlorophyll | 4.625 |
| Ascorbic acid | 3.247 |
| Phytohormones (g g^−1^ DW): |  |
| Indole-3-acetic acid | 0.873 |
| Gibberellins | 0.802 |
| Zeatin | 0.936 |
| Abscisic acid | 0.292 |
